# Supplementary figures and images for: Tissue-specific degradation of essential centrosome components reveals distinct microtubule populations at microtubule organizing centers
Source: PLoS Biol. 2018 Aug 6;16(8):e2005189. doi: 10.1371/journal.pbio.2005189 (PMC6103517; doi:10.1371/journal.pbio.2005189)

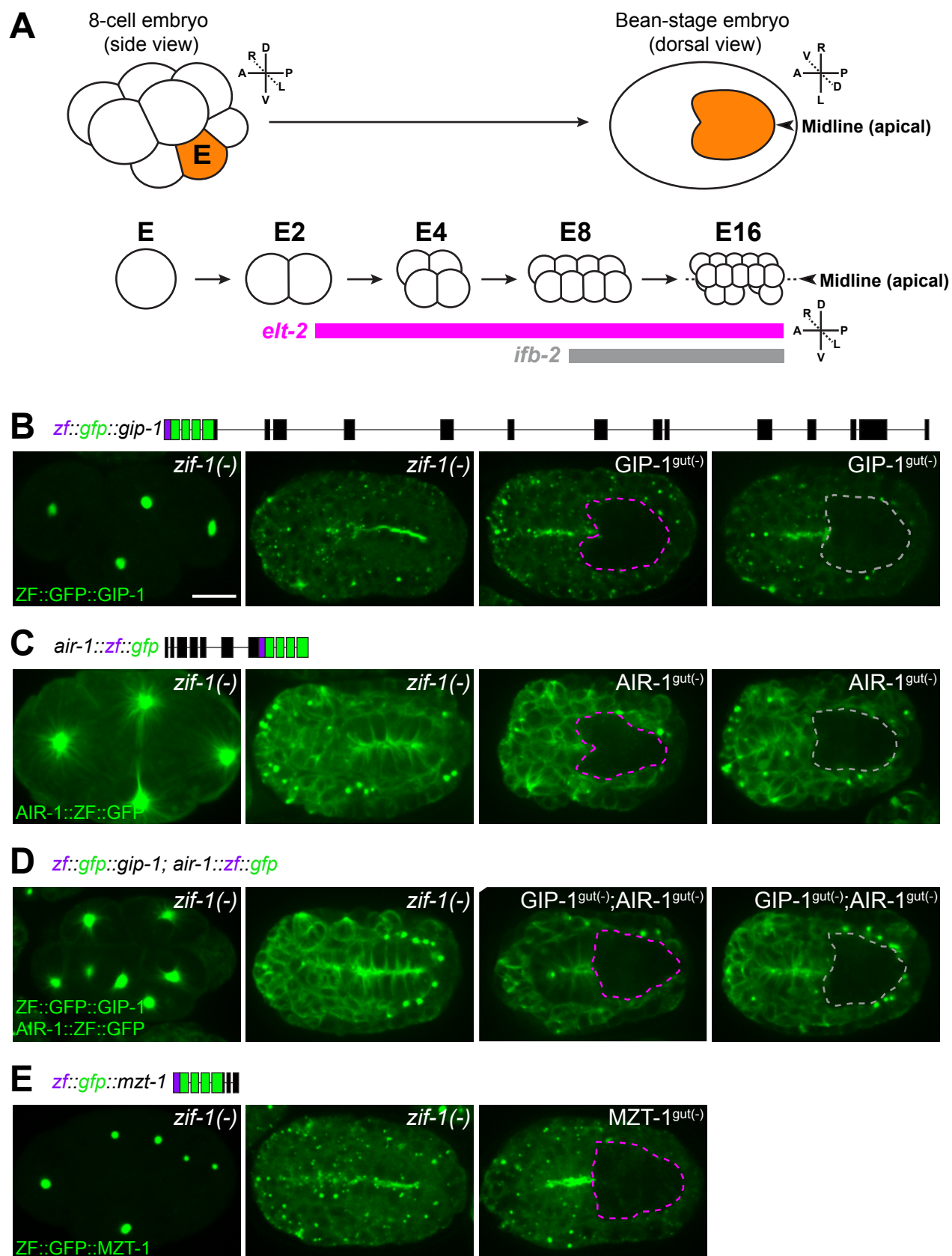

Figure S1

Supplement: S1 Fig — (A) Cartoon of intestinal development. The “E” blastomere is specified at the eight-cell stage of the embryo and undergoes four rounds of divisions to give rise to the E16 intestinal primordium. At E16 (the approximately 250-cell stage of the embryo), intestinal cells are arranged into two tiers and polarize around a central midline. At “bean stage,” all intestinal cells are polarized with their apical surfaces facing the midline. The elt-2 promoter is active beginning in E2–E4 and the ifb-2 promoter around E8. (B–E) CRISPR tagging of endogenous GIP-1 (B, D), AIR-1 (C, D), and MZT-1 (E) with ZF (purple) and GFP (green). All proteins localize to the centrosome in dividing cells (first panel) and to the apical ncMTOC in the polarized intestinal primordium (second panel). Note that AIR-1 also decorates microtubules. Each protein is efficiently degraded after ZIF-1 expression from the elt-2 promoter (third panel, magenta) or the ifb-2 promoter (fourth panel, gray). CRISPR, Clustered Regularly Interspaced Short Palindromic Repeats; GFP, Green Fluorescent Protein; ncMTOC, non-centrosomal microtubule organizing center; ZF, zinc finger domain 1. (PDF) [file pbio.2005189.s001.pdf]

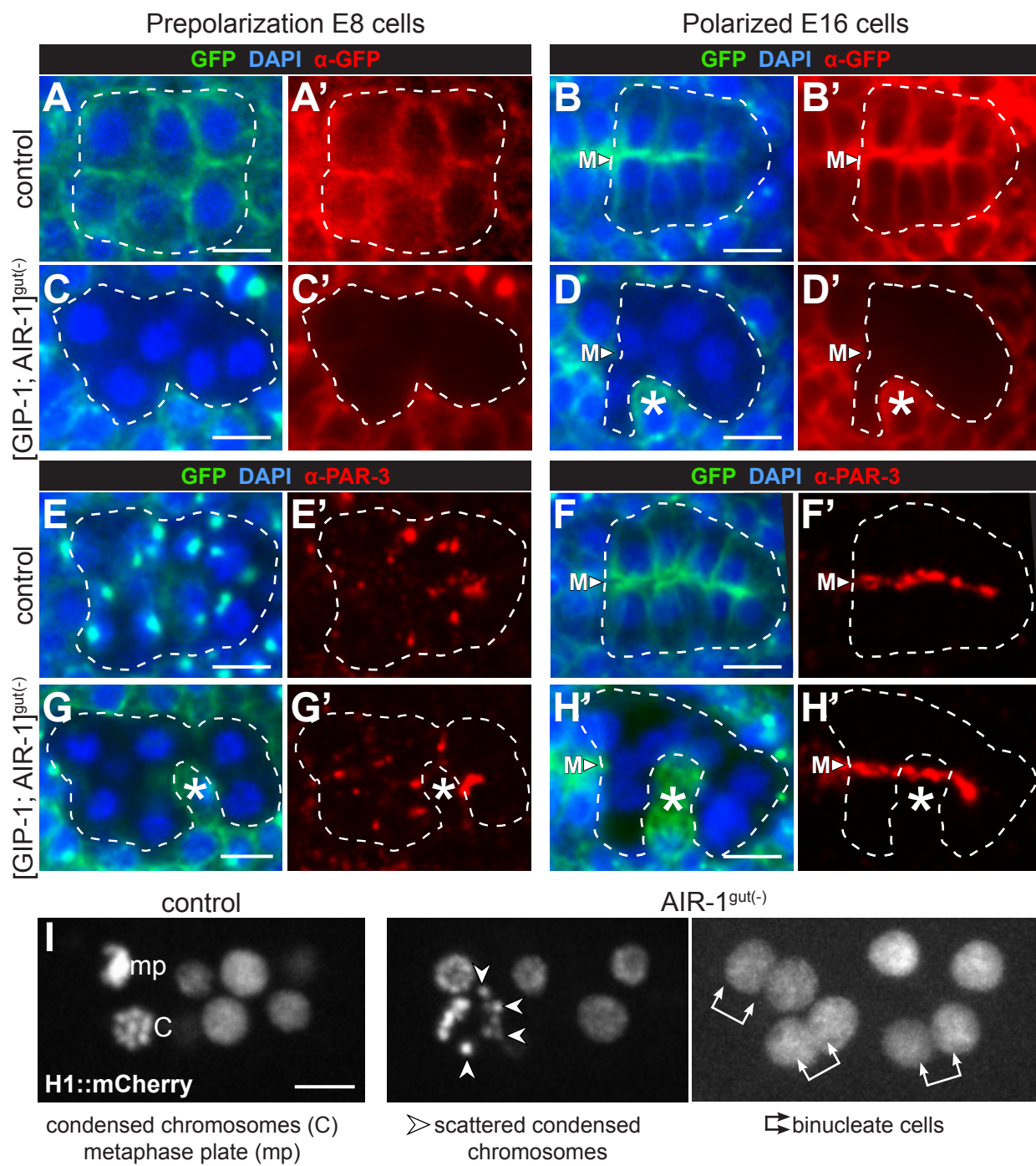

**Figure S2**

Supplement: S2 Fig — (A–D) Antibody staining of strain JLF232 for GFP shows that ZF::GFP::GIP-1 and AIR-1::ZF::GFP co-depletion is highly efficient. In control embryos, which lack ZIF-1, perduring GFP signal (green, A, B) and α-GFP antibody staining (red, A′, B′) from ZF::GFP::GIP-1 and AIR-1::ZF::GFP are observed in E8 (A) and polarized E16 (B) intestinal primordia. In embryos carrying the ifb-2p::zif-1 transgene, localized intestinal GFP was not observed for either perduring GFP (C, D) or α-GFP antibody staining (C′, D′). (E–H) PAR-3 (red) is localized as discrete puncta in the E8 intestinal primordium prior to polarization in both control (E′) and GIP-1;AIR-1 co-depleted embryos (G′). In the polarized E16 intestinal primordium, PAR-3 localizes to the apical midline (“M”) in both control (F′) and GIP-1;AIR-1 co-depleted (H′) embryos. Asterisks indicate primordial germ cell fluorescence. A dashed white line outlines the E8 and the polarized E16 intestinal primordia. DNA is labeled with DAPI in (A–H). (I) In control embryos, nuclear number and position is normal, and condensed prophase and metaphase chromosomes remain close together (left panel). By contrast, in addition to nuclear number defects (Fig 2), some AIR-1gut(−) embryos have scattered condensed chromosomes (white arrowheads) and binucleate cells (white joined arrows). We note that all of the scattered condensed chromosomes in the middle panel eventually segregate into two distinct groups. GFP, Green Fluorescent Protein; ZF, zinc finger domain 1; α-GFP, anti-GFP antibody. (PDF) [file pbio.2005189.s002.pdf]

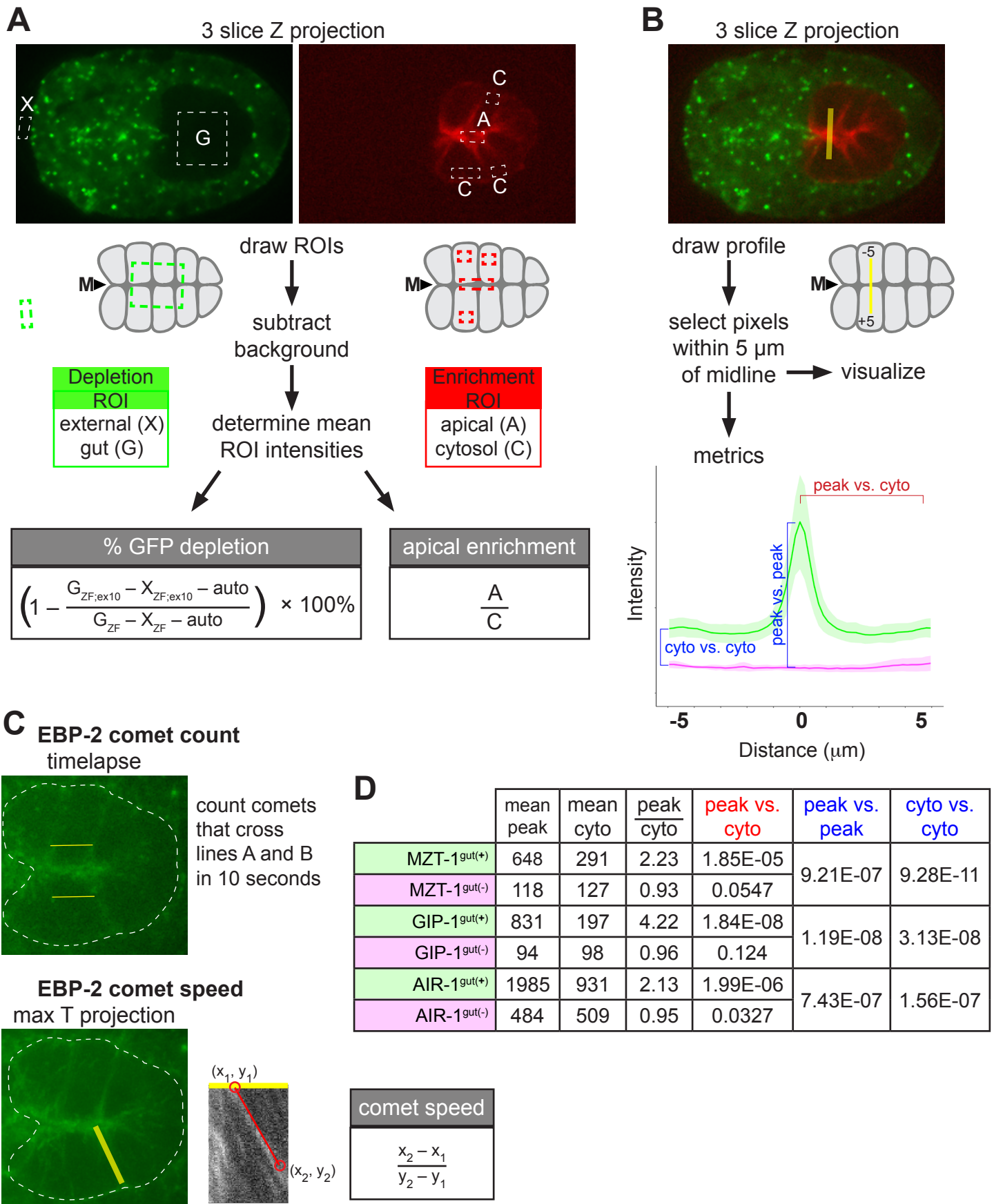

Figure S3

Supplement: S3 Fig — (A) Fluorescence intensity measurements and calculations for GFP depletion and apical enrichment. Left: percent GFP depletion. External (X) and gut (G) ROIs were drawn in the green channel, and percent GFP depletion was determined by comparing the intestinal GFP signal in “ZF;ex10” depletion embryos to “ZF” control embryos. Right: apical enrichment. Apical (A) and cytosolic (C) ROIs were defined in the red channel for α-tubulin (Fig 6) and EBP-2 (Fig 7), and the signal intensity in those ROIs was used to calculate apical enrichment for mCherry::TBA-1 and EBP-2::GFP, as shown. Example ROIs are shown. (B) Fluorescence intensity profile plots. A 1-μm-wide line was drawn across the midline in each embryo analyzed and pixels within 5 μm of the midline were selected to generate a line profile. To quantify GFP depletion in degradation strains, peak and cytoplasmic regions of the profiles were defined as in Materials and methods and compared in (D). Mean profiles for control MZT-1gut(+) (ZF::GFP::MZT-1, green) and MZT-1gut(−) (magenta) intestinal primordia are shown. (C) EBP-2 comet speed and number measurements. The relative number of EBP-2 comets originating from the midline was estimated by counting comets that crossed 5-μm lines drawn 3 μm from either side of the midline over the course of 10 seconds. Comet speed was measured by generating kymographs and measuring the slope of the comets. An example of a MZT-1gut(−) image is shown; top: single time frame with lines for comet counting; bottom: max time-projection with one kymograph line drawn (left: yellow line) and the resulting kymograph indicating the measured slope of an example comet (right: red line, speed). (D) Profile metrics for indicated genotypes to calculate GFP depletion. Within-genotype comparison (red): For all gut(−) embryos, the cytoplasmic intensity value is slightly higher than the peak value, likely due to out-of-focus light from non-degraded ZF::GFP in surrounding tissues. For all embryos without deg [file pbio.2005189.s003.pdf]

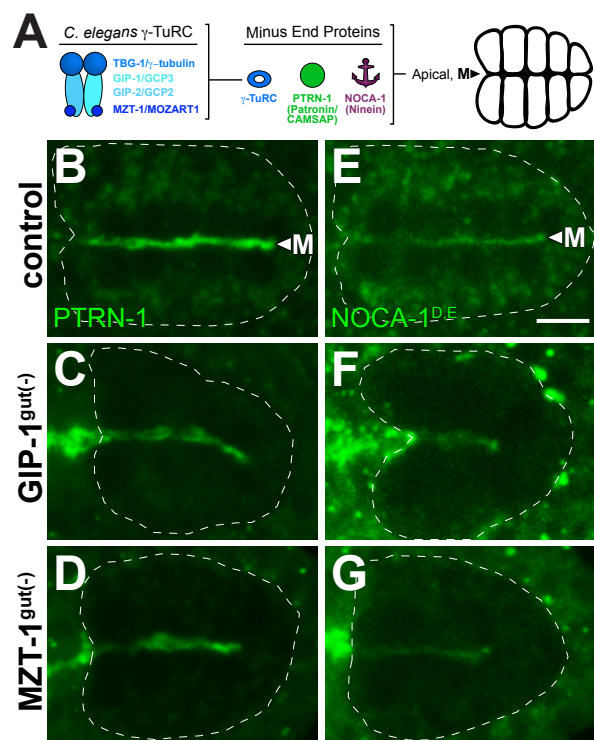

**Figure S4**

Supplement: S4 Fig — Cartoon of γ-TuRC and other minus-end regulators found at the apical ncMTOC. Images are projected optical sections through the midline of live bean-stage embryos. The intestinal primordium is outlined by white dashed lines. (B–D) Endogenously tagged PTRN-1::GFP localizes apically in control (B, n = 8/8), GIP-1gut(−) (C, n = 22/24), and MZT-1gut(−) (D, n = 18/19) embryos. (E–G) A single-copy insertion NOCA-1(de)::GFP transgene localizes apically in control (E, n = 25/25), GIP-1gut(−) (F, n = 13/13), and MZT-1gut(−) (G, n = 16/16) embryos. GFP, Green Fluorescent Protein; ncMTOC, non-centrosomal microtubule organizing center; γ-TuRC, γ-tubulin ring complex. (PDF) [file pbio.2005189.s004.pdf]

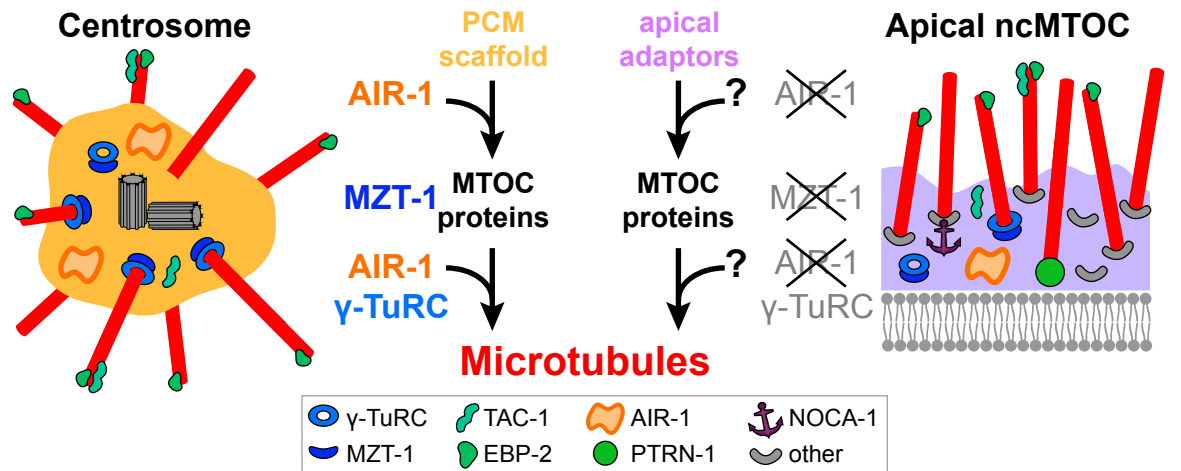

**Figure S5**

Supplement: S5 Fig — A summary of the factors localized to and required for the growth and localization of microtubules at two different MTOCs, the centrosome and the apical surface of intestinal epithelial cells. AIR-1 is required to localize MTOC proteins GIP-1 and TAC-1 to the centrosome but not to the apical ncMTOC. MZT-1 is required to localize GIP-1 to the centrosome but not to the apical ncMTOC. GIP-1 is required for localization of all other γ-TuRC components to both MTOCs. AIR-1 and GIP-1 are not required to organize apical microtubules. However, GIP-1 (but not MZT-1) is required to build a subset of dynamic microtubules and for normal microtubule growth speeds at the apical ncMTOC. These findings suggest that other unknown factors (gray) are redundantly required at the apical ncMTOC for microtubule nucleation and organization. MTOC, microtubule organizing center; ncMTOC, non-centrosomal microtubule organizing center; γ-TuRC, γ-tubulin ring complex. (PDF) [file pbio.2005189.s005.pdf]
