# Supplementary material for: Tissue-specific degradation of essential centrosome components reveals distinct microtubule populations at microtubule organizing centers
Source: PLoS Biol. 2018 Aug 6;16(8):e2005189. doi: 10.1371/journal.pbio.2005189 (PMC6103517; doi:10.1371/journal.pbio.2005189)
Supplement: S1 Text — (RTF) [file pbio.2005189.s008.rtf]

CLUSTAL O(1.2.4) multiple sequence alignment


C.elegans_MZT-1/W03G9.8      -----------------MSDPKKHTQRIVEMGKFL 
C.japonica_CJA02437          -----------------MNDSRKSPQLVMELAKFL
C.briggsae_BP:CBP44147       -----------------MSELKKDTQQAMEVAKFL 
C.remanei_RP:RP31060         ----------------------------MEMAKFL  
Drosophila_MZT1              -----------MSEQPTQHKSDDRFTILQTLSDVV 
Arabidopsis_GIP1             ------------MDEEASRTARESLELVFRMSNIL 
Human_MZT1                   MASSSGAGAAAAAAAANLNAVRETMDVLLEISRIL 
S.pombe_MZT1                 ----------------MSESTKETIEVLYEIGTLL 
                                                           :. .:

C.elegans_MZT-1/W03G9.8      NVFLTAEQVSSVERLLSLGVSPLNLVRLIQNLGTP 
C.japonica_CJA02437          NVFLTAEQIAAVEKMLALGVTPVNLVRLIQNLGSS 
C.briggsae_BP:CBP44147       NVFLTIDQIVAVEKLLAMGVSPINLVNFLRRVD-G 
C.remanei_RP:RP31060         NVFLTVEQVVAVEKLLAIGVPPINIVRLLQSVS-P 
Drosophila_MZT1              DSGLSKEALKICIELVDNGVCGGALAHVIRTIREE 
Arabidopsis_GIP1             DTGLDRHTLSVLIALCDLGVNPEALATVVKELRRE 
Human_MZT1                   NTGLDMETLSICVRLCEQGINPEALSSVIKELRKA 
S.pombe_MZT1                 GTELDKTTLSLCISLCENNVHPEAIAQIIREIRMA 
                             .  *    :     :   .:    :  .:: :                          

C.elegans_MZT-1/W03G9.8      STQSS----PSNRENALS-----	67 
C.japonica_CJA02437          STLNS----PSNRENSLQ-----	67
C.briggsae_BP:CBP44147       STQGS----PSNRENAL------	65 
C.remanei_RP:RP31060         NQQMS----PGNRENGI------	54 
Drosophila_MZT1              IQDDEDKESDDSGESAASTDSTL	82 
Arabidopsis_GIP1             SIPDSVTTTPSIH----------	71 
Human_MZT1                   TEALKA-AENMTS----------	82 
S.pombe_MZT1                 QEQTVD-TEPS------------	64
